# Antibodies Cross-Reactive with Bundibugyo Virus in Ferrets Vaccinated with Ebola Virus Vaccine

**DOI:** 10.3201/eid3208.260948

**Published:** 2026-08

**Authors:** Jordan Wight, Helene Schulz, Logan Banadyga

**Affiliations:** Public Health Agency of Canada, Winnipeg, Manitoba, Canada (J. Wight, H. Schulz, L. Banadyga); University of Manitoba, Winnipeg (L. Banadyga)

**Keywords:** Ebola virus, viruses, Bundibugyo virus, Sudan virus, Filovirus, orthoebolavirus, rVSV-EBOV, ERVEBO, rVSV-SUDV, vaccine, cross-protection, ferrets, vaccine-preventable diseases

## Abstract

Banked serum samples from ferrets previously immunized with the Ebola virus vaccine revealed a prominent but limited humoral immune response that cross-reacted with Bundibugyo virus. The supporting immunogenicity data we report may help guide the ongoing response to the current outbreak of Bundibugyo virus in the Democratic Republic of the Congo.

On May 15, 2026, the Democratic Republic of the Congo (DRC) declared an outbreak of Ebola disease after laboratory confirmation of persons infected with Bundibugyo virus (BDBV) in the country’s Ituri Province (*1*). At the time, DRC reported 246 suspected cases and 65 deaths associated with an outbreak that probably began weeks earlier. Since the declaration, the outbreak has expanded substantially; 695 confirmed cases and 138 deaths have been reported as of June 13, including 19 cases and 2 deaths in neighboring Uganda (*2*). On May 16, the World Health Organization classified the outbreak as a Public Health Emergency of International Concern. This outbreak marks only the third known emergence of BDBV, which previously caused outbreaks in Uganda in 2007 and in DRC in 2012.

Currently, no therapeutics or vaccines are available to treat or prevent BDBV disease. Although countermeasures are available for the closely related Ebola virus (EBOV), their efficacy against BDBV is unclear. Of particular interest is the recombinant vesicular stomatitis virus (rVSV)–based vaccine encoding the EBOV glycoprotein (rVSV-EBOV) (tradename ERVEBO; Merck, https://www.merck.com), which has been approved by the US Food and Drug Administration and the European Medicines Agency for prevention of EBOV disease (EVD). A previous study by Falzarano et al. (*3*) showed that 3 of 4 cynomolgus macaques vaccinated with rVSV-EBOV were protected against subsequent challenge with BDBV. Conversely, a separate study by Mire et al. (*4*) demonstrated that cynomolgus macaques vaccinated with a blend of rVSV-EBOV and an analogous Sudan virus (SUDV) vaccine (rVSV-SUDV) protected only 1 of 3 animals from BDBV challenge, although a prime dose of rVSV-SUDV followed by a booster dose of rVSV-EBOV protected 3 of 3 animals. To help establish whether cross-reactive immune responses against BDBV can be elicited by heterologous rVSV-based vaccines, we sought to evaluate the levels of BDBV-specific IgG in the context of the ferret model (*5*).

We collected serum samples from ferrets 27 days after vaccination with rVSV-EBOV or rVSV-SUDV and quantified EBOV, SUDV, and BDBV glycoprotein-specific IgG levels by using ELISA (Appendix). Both rVSV-EBOV and rVSV-SUDV elicited strong humoral responses to their homologous antigens; serum from all animals exhibited maximum absorbance at dilutions of 1:400 (Figure 1). As expected, the animals survived subsequent challenge with a uniformly lethal dose of the corresponding virus (J. Wight, H. Schulz, L. Banadyga, unpub. data). Serum samples from ferrets vaccinated with rVSV-EBOV exhibited moderate levels of absorbance against BDBV glycoprotein, in contrast with serum samples from animals vaccinated with rVSV-SUDV, which exhibited very low levels of absorbance (Figure 1). Since those data suggested that vaccination with rVSV-EBOV—but not rVSV-SUDV—elicits a heterologous immune response to BDBV, we further quantified the levels of glycoprotein-specific IgG in the serum from animals vaccinated with rVSV-EBOV by endpoint titration (Figure 2). Reciprocal endpoint titers for EBOV glycoprotein-specific IgG ranged from 25,600 to 76,800, with a geometric mean of 56,554. At 16,582, the geometric mean endpoint titer for BDBV glycoprotein-specific IgG was significantly lower than that for EBOV glycoprotein (Wilcoxon statistical test = 3; p = 1.6 × 10^–4^), despite limited overlap in the range of titers, which extended from 3,200 to 30,270. We detected no EBOV- or BDBV-specific IgG in the control animals.

**Figure 1:**
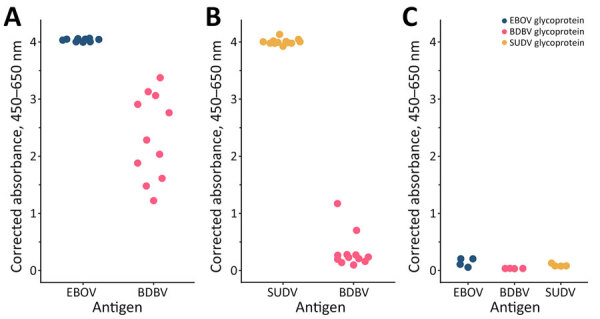
Detection of EBOV glycoprotein–, BDBV glycoprotein–, and SUDV glycoprotein–specific IgG in rVSV-EBOV– and rVSV-SUDV–vaccinated ferrets and controls in study of antibodies cross-reactive with BDBV in ferrets vaccinated with EBOV vaccine. Ferrets were vaccinated with 2 × 10^5^ plaque-forming units of either rVSV-EBOV (A) or rVSV-SUDV (B). Control animals received saline only (C). On day 27 postvaccination, serum samples were collected. Serum was diluted 1:400 and assayed by indirect ELISA. Data shown are corrected absorbance levels calculated by subtracting the absorbance at 650 nm from the absorbance at 450 nm. BDBV, Bundibugyo virus; EBOV, Ebola virus; rVSV-EBOV, recombinant vesicular stomatitis virus–based vaccine encoding the Ebola virus glycoprotein; rVSV-SUDV, recombinant vesicular stomatitis virus–based vaccine encoding the Sudan virus glycoprotein.

**Figure 2:**
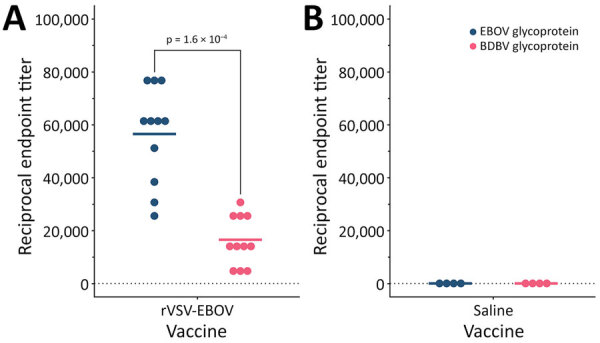
Endpoint titers of EBOV glycoprotein– and BDBV glycoprotein–specific IgG in rVSV-EBOV– vaccinated ferrets (A) and controls (B) in study of antibodies cross-reactive with BDBV in ferrets vaccinated with EBOV vaccine. Serial dilutions of serum were assayed by indirect ELISA. Date shown are reciprocal endpoint titers exceeding the cut-off value of the mean plus 3 times the SD of the 1:400 dilution of prevaccination serum collected from all ferrets. Horizontal colored lines represent the mean reciprocal endpoint titers. The dashed line at a reciprocal endpoint titer of 400 represents the lower limit of detection of the assay. BDBV, Bundibugyo virus; EBOV, Ebola virus; rVSV-EBOV, recombinant vesicular stomatitis virus–based vaccine encoding the Ebola virus glycoprotein.

The macaque studies by Falzarano et al. (*3*) and Mire et al. (*4*) suggest that BDBV cross-protection from rVSV-EBOV vaccination may be possible in some cases; however, apparently conflicting results, combined with different vaccination schemes, small group sizes, and a model system that is not uniformly lethal, make meaningful conclusions hard to draw. Thus, those studies do not provide conclusive evidence either for or against cross-protection. Although our data do not definitively answer the questions regarding the ability of rVSV-EBOV to confer protection against BDBV, they do suggest a mechanistic foundation that might underlie cross-protection. Because nonneutralizing antibodies are key to rVSV-EBOV–mediated protection from EVD (*6*,*7*), a cross-reactive humoral immune response probably is a prerequisite for any potential BDBV cross-protection that this vaccine might confer. Future work by our group will directly assess the ability of rVSV-EBOV to elicit cross-protection against a uniformly lethal dose of BDBV in ferrets. We are particularly interested in understanding whether a homologous or heterologous vaccine boost may improve cross protection, given our data suggesting that rVSV-SUDV vaccination produces a meager cross-reactive response and data from Mire et al. demonstrating that rVSV-SUDV vaccination followed by rVSV-EBOV boost resulted in complete cross-protection (*4*).

Although limited in scope, our analysis of cross-reactive antibodies in vaccinated ferrets provides further evidence that rVSV-EBOV vaccination elicits BDBV-specific antibodies, extending observations that have already been made in humans (*8*). In the midst of an ongoing public health response against BDBV, this study provides supportive, albeit not conclusive, immunogenicity evidence that may be critical in determining the potential utility of the rVSV-EBOV vaccine, which was already administered to >200,000 persons in DRC during the 2018–2020 EVD outbreak (*9*).

Appendix Additional information about antibodies cross-reactive with Bundibugyo virus in ferrets vaccinated with Ebola virus vaccine.
