# Supplementary material for: Antibodies Cross-Reactive with Bundibugyo Virus in Ferrets Vaccinated with Ebola Virus Vaccine
Source: Emerg Infect Dis. 2026 Aug;32(8):1360–3. doi: 10.3201/eid3208.260948 (PMC13426813; doi:10.3201/eid3208.260948)
Supplement: Appendix — Additional information about antibodies cross-reactive with Bundibugyo virus in ferrets vaccinated with Ebola virus vaccine. [file 26-0948-Techapp-s1.pdf]

Article DOI: https://doi.org/10.3201/eid3208.262948

EID cannot ensure accessibility for supplementary materials supplied by authors. Readers who have difficulty accessing supplementary content should contact the authors for assistance.

Antibodies Cross-Reactive with Bundibugyo Virus in Ferrets Vaccinated with Ebola Virus Vaccine

Appendix

Animal Ethics and Biosafety Statement

Animal work was conducted at the Canadian Science Centre for Human and Animal Health (CSCHAH), National Microbiology Laboratory of the Public Health Agency of Canada in Winnipeg, Manitoba. Animal use protocols were approved by the Institutional Animal Care Committee in accordance with guidelines from the Canadian Council on Animal Care.

Cells and Viruses

Vero E6 (ATCC CRL-1586) cells were maintained in DMEM (ThermoFisher Scientific, Cat # 11965092) supplemented with 10% heat-inactivated fetal bovine serum (FBS; Corning, Cat # 35-077-CV), 100 U/mL of penicillin and 100 µg/mL streptomycin (ThermoFisher Scientific, Cat # 15140122), and 2 mM L-glutamine (ThermoFisher Scientific, Cat # 25030081). rVSVΔG-EBOV GP variant Kikwit (i.e., rVSV-EBOV) and rVSVΔG-SUDV GP variant Boneface (i.e., rVSV-SUDV) were recovered as previously described (*1*). Viruses were grown and titered on Vero E6 cells.

Ferret Vaccination

Ferrets (*Mustela putorius furo*) were purchased from Marshall BioResources and acclimatized for at least seven days before study commencement. All animals were monitored at least daily and were provided food and water *ad libitum*. All invasive manipulations, such as blood collection and vaccination, were performed under isoflurane anesthesia.

Equal numbers of male and female ferrets were randomly assigned to vaccination and control groups. Animals were vaccinated intramuscularly with either rVSV-EBOV (n = 12) or rVSV-SUDV (n = 12) at a dose of 2 x 10^5 PFU, diluted in 0.9% saline to a total volume of 500 µL, while control animals (n = 4) received 500 µL of 0.9% saline. Vaccinations were conducted over the course of two separate studies, with the group sizes split evenly between them. For the first study, animals received a single injection of vaccine in one quadriceps muscle, while for the second study, the vaccine was administered in two injections divided equally between the two quadriceps muscles. Blood was collected from the jugular vein immediately prior to vaccination and on day 27 post-vaccination and transferred to tubes containing serum separation gel (Sarstedt Inc, Cat # 41.1378.005). Serum separation tubes were spun at 8,000 x g for five minutes, after which serum was removed and subsequently stored at -80°C until analysis. For the present analysis, we lacked sufficient serum volume from one animal belonging to the second study to conduct the BDBV GP ELISAs. For this reason, the total number of samples from rVSV-EBOV vaccinated animals is 11.

IgG ELISAs

EBOV GP and SUDV GP specific IgG levels were determined in serum samples collected immediately prior to vaccination and on day 27 post-vaccination by indirect ELISA. Briefly, half-area high-binding 96-well assay plates (Corning, Cat # 3690) were coated using recombinant transmembrane domain-deleted glycoproteins, specifically EBOV (IBT Bioservices, Cat # 0501-001), SUDV variant Boneface (IBT Bioservices, Cat # 0502-001), or BDBV (IBT Bioservices, Cat # 0505-015). Recombinant proteins were prepared in 50 mM carbonate-bicarbonate buffer pH 9.6 at a concentration of 1 µg/mL. Coating was performed by adding 30 µL of this solution to each well and incubating overnight at 4°C. The following day, the coating solution was removed and wells were blocked with 100 µL of 5% skim milk prepared in phosphate-buffered saline (PBS) pH 7.4 for 1 hour at 37°C. After removing the blocking solution, 30 µL of serum samples diluted in 2% skim milk in PBS were added in triplicate wells and incubated for 1 hour at 37°C. Plates were washed four times with 0.1% Tween-20 in PBS, and 30 µL of anti-ferret IgG (H+L) HRP secondary antibody (Novus Biologicals, Cat # NB7224) diluted 1:10,000 in 2% milk was added per well. Plates were washed again and 50 µL of 3,3’,5,5’-tetramethylbenzidine (TMB; Invitrogen, Cat # 002023) was added

and incubated in the dark for 30 minutes. Color development was stopped by adding 50 µL of 0.16 M sulfuric acid.

Initially, all serum samples were diluted at 1:400 and absorbance was measured using a Varioskan LUX Microplate Reader (Thermo Scientific). Absorbance at 450 nm minus the absorbance at 650 nm was used to calculate the corrected absorbance. To determine endpoint titers, samples from rVSV-EBOV vaccinated and control animals were tested in triplicate at the following dilutions: 1:400, 1:600, 1:800, 1:1,200, 1:1,600, 1:2,400, 1:3,200, 1:4,800, 1:6,400, 1:9,600, 1:12,800, 1:15,360, 1:19,200, 1:25,600, 1:30,720, 1:38,400, 1:51,200, 1:61440, and 1:76,800. Positivity cut-off values were established as the mean plus three times the standard deviation of a 1:400 dilution of pre-vaccination serum collected from all ferrets. Endpoint dilution titers were determined as the highest dilution that exceeded the cut-off value.

Statistical Analysis and Data Visualization

R version 4.4.3 (R Project for Statistical Computing, https://www.r-project.org) was used to perform data manipulation, statistical analysis, and data visualization. A p-value < 0.05 was considered as significant for all tests. To compare reciprocal endpoint titers between EBOV GP and BDBV GP antigens for rVSV-EBOV vaccinated ferrets, a Wilcoxon (W) rank-sum test with continuity correction was performed. R packages used included ggbeeswarm version 0.7.3 (https://CRAN.R-project.org/package=ggbeeswarm), ggplot2 version 4.0.2 (https://ggplot2.tidyverse.org), ggtext version 0.1.2 (https://CRAN.R-project.org/package=ggtext), and scales version 1.4.0 (https://CRAN.R-project.org/package=scales).

Reference

1. Garbutt M, Liebscher R, Wahl-Jensen V, Jones S, Möller P, Wagner R, et al. Properties of replication-competent vesicular stomatitis virus vectors expressing glycoproteins of filoviruses and arenaviruses. J Virol. 2004;78:5458–65. PubMed https://doi.org/10.1128/JVI.78.10.5458-5465.2004
